# Dietary Effects on Monocyte Phenotypes in Subjects With Hypertriglyceridemia and Metabolic Syndrome

**DOI:** 10.1016/j.jacbts.2023.02.011

**Published:** 2023-04-26

**Authors:** Zeqin Lian, Xiao-Yuan Dai Perrard, Antu Kalathookunnel Antony, Xueying Peng, Lu Xu, Jing Ni, Bingqian Zhang, Veronica O’Brien, Anum Saeed, Xiaoming Jia, Aliza Hussain, Bing Yu, Scott I. Simon, Frank M. Sacks, Ron C. Hoogeveen, Christie M. Ballantyne, Huaizhu Wu

**Affiliations:** a Department of Medicine, Baylor College of Medicine, Houston, Texas, USA; b Department of Clinical Pharmacy, Key Laboratory of Clinical Cancer Pharmacology and Toxicology Research of Zhejiang Province, Affiliated Hangzhou First People’s Hospital, Zhejiang University School of Medicine, Hangzhou, Zhejiang, People’s Republic of China; c Heart and Vascular Institute, University of Pittsburgh Medical Center, Pittsburgh, Pennsylvania, USA; d Center for Cardiometabolic Disease Prevention, Baylor College of Medicine, Houston, Texas, USA; e Department of Epidemiology, Human Genetics and Environmental Sciences, School of Public Health, University of Texas Health Science Center, Houston, Texas, USA; f Department of Biomedical Engineering, University of California, Davis, California, USA; g Department of Nutrition, Harvard T.H. Chan School of Public Health, and Department of Medicine, Harvard Medical School and Brigham and Women’s Hospital, Boston, Massachusetts, USA

**Keywords:** ASCVD, high saturated fat diet, hypertriglyceridemia, low saturated fat diet, monocyte

## Abstract

In patients with hypertriglyceridemia, a short-term low–saturated fat vs high–saturated fat diet induced lower plasma lipids and improved monocyte phenotypes. These findings highlight the role of diet fat content and composition for monocyte phenotypes and possibly cardiovascular disease risk in these patients. (Effects of Dietary Interventions on Monocytes in Metabolic Syndrome; NCT03591588)

## Highlights

• Hypertriglyceridemia and metabolic syndrome increase the risk for atherosclerotic cardiovascular disease. Dietary composition may affect plasma levels of triglycerides and therefore the risk for atherosclerotic cardiovascular disease.
• In patients with hypertriglyceridemia and metabolic syndrome, a short-term randomized LSFD vs HSFD induced lower plasma levels of postprandial triglyceride and LDL-triglyceride and fasting and postprandial total cholesterol and LDL cholesterol, decreased monocyte intracellular lipid accumulation, and reduced monocyte adhesion and oxidized LDL uptake.
• These findings highlight the importance of dietary fat content and composition for monocyte phenotypes and possibly atherosclerotic cardiovascular disease risk in patients with hypertriglyceridemia and metabolic syndrome.

## Visual Abstract

**Figure:**
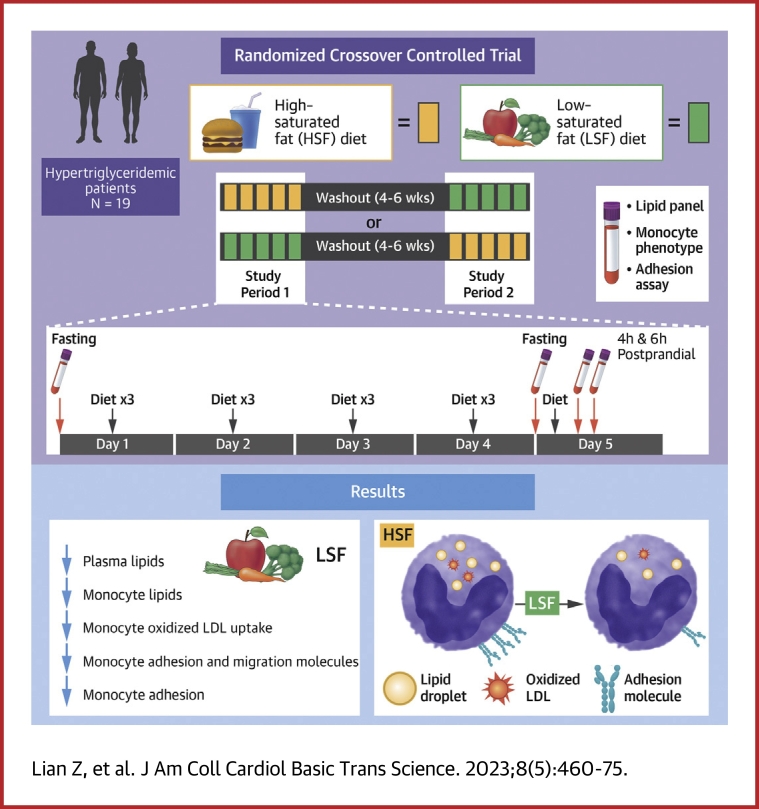

Hypertriglyceridemia with metabolic syndrome is causally associated with the development of atherosclerotic cardiovascular disease (ASCVD).^1^, ^2^, ^3^, ^4^, ^5^ The mechanisms linking hypertriglyceridemia and metabolic syndrome with ASCVD risk are not fully understood but may include inflammation, which has been implicated in ASCVD and correlates with hypertriglyceridemia and metabolic syndrome.^2^^,^^5^, ^6^, ^7^, ^8^, ^9^, ^10^, ^11^, ^12^, ^13^ Diet intervention is an important strategy for ASCVD prevention, and changes in diet composition may affect ASCVD risk.^14^ However, the method by which immune cells respond to different dietary compositions and may subsequently affect ASCVD risk is still ill defined.

Monocytes, one of the most important immune cell types in the circulation, respond to changes in plasma lipid profiles and play pivotal roles in atherosclerosis.^15^ In humans, hypertriglyceridemia with metabolic syndrome or postprandial hypertriglyceridemia induced by a single high–saturated fat meal increases lipid accumulation within monocytes, causing formation of foamy monocytes, which contain intracellular lipid droplets and exhibit inflammatory phenotypes with enhanced adhesion to endothelium.^16^, ^17^, ^18^, ^19^, ^20^ In mouse models of atherosclerosis, a high–saturated fat, high-cholesterol diet induces early formation of circulating foamy monocytes, which infiltrate into arterial walls and contribute to the development of atherosclerosis.^21^^,^^22^ In contrast, replacing saturated fat with unsaturated fat in the diet results in decreased formation of foamy monocytes, which correlates with reduced monocyte adhesion and decreased atherosclerotic plaque formation.^23^ These observations in mouse models of atherosclerosis suggest that dietary composition may affect atherosclerosis development by regulating foamy monocyte formation and phenotypes.

More than one-third of Americans have metabolic syndrome, which is present in almost one-half the population at age 60 years and has been increasing in younger adults.^24^ Diets high in fat and protein but low in carbohydrates have gained increasing popularity and may help reduce body weight in patients with metabolic syndrome.^14^^,^^25^ How monocytes respond to changes in dietary composition in humans with hypertriglyceridemia and metabolic syndrome and may therefore affect ASCVD risk remains to be determined.

To investigate the role of diet composition in lipid and inflammatory responses in hypertriglyceridemia with metabolic syndrome, we examined the effects of a short-term low–saturated fat diet (LSFD) vs high–saturated fat diet (HSFD) on plasma lipids, monocyte phenotypes, and monocyte adhesion in subjects with hypertriglyceridemia and metabolic syndrome.

## Methods

### Study Design and Participants

The study used a randomized crossover design.

Nineteen adults with hypertriglyceridemia and metabolic syndrome (Table 1) were recruited. Inclusion criteria were hypertriglyceridemia (fasting triglyceride levels ≥150 mg/dL) plus at least 2 of the following 4 criteria: 1) abdominal obesity (ie, waist circumference >88 cm for women and >102 cm for men); 2) high-density lipoprotein cholesterol (HDL-C) level <50 mg/dL for women and <40 mg/dL for men; 3) glucose levels ≥100 mg/dL; and 4) blood pressure ≥130/85 mm Hg or a history of hypertension. Exclusion criteria were pregnancy or breastfeeding, current smoking, obvious inflammation or other acute illnesses, obesity due to endocrinologic disorders (including polycystic ovary syndrome and partial lipodystrophy), diabetes requiring insulin or >1 oral medication, myocardial infarction or any hospitalization within the past 2 months, fasting triglyceride level ≥650 mg/dL, history of pancreatitis, or current steroid use.

**Table 1:** Baseline Characteristics of Participants (N = 19)

Demographic characteristics	
Age, y	53.9 ± 3.20
Male	10 (52.6)
Physical examination	
Weight, lb	219.0 ± 8.00
BMI, kg/m^2^	37.2 ± 2.57
SBP, mm Hg	130.9 ± 3.42
DBP, mm Hg	78.9 ± 2.23
Laboratory values	
Cholesterol, mg/dL	200.7 ± 8.26
Triglyceride, mg/dL	232.1 ± 26.4
LDL-C, mg/dL	126.4 ± 8.55
HDL-C, mg/dL	42.4 ± 1.7
Glucose, mg/dL	88.0 ± 8.31
HbA_1c_, %	6.6 ± 0.35
hsCRP, mg/L	2.6 (0.86, 10.3)

Values are mean ± SEM, n (%), or median (Q1, Q3).

BMI = body mass index; DBP = diastolic blood pressure; HbA_1c_ = hemoglobin A1c; HDL-C = high-density lipoprotein cholesterol; hsCRP = high-sensitivity C-reactive protein; LDL-C = low-density lipoprotein cholesterol; SBP = systolic blood pressure.

The sample size was determined by power calculation based on the HSFD test meal–induced postprandial changes in monocyte lipid accumulation (the primary outcome in the current study) that we observed in subjects with metabolic syndrome in our previous study.^18^ The mean ± SD of the primary outcome at 5 hours postprandially compared with fasting was 1.1 ± 1.0 AU. Based on an assumption of 70% of the HSFD test meal–induced postprandial changes in our primary outcome achieved in the current study, 19 subjects would give us ∼89% power to detect the difference at a SD of 1.0 and an α level of 0.05.

### Diets and Study Procedures

**Figure 1:**
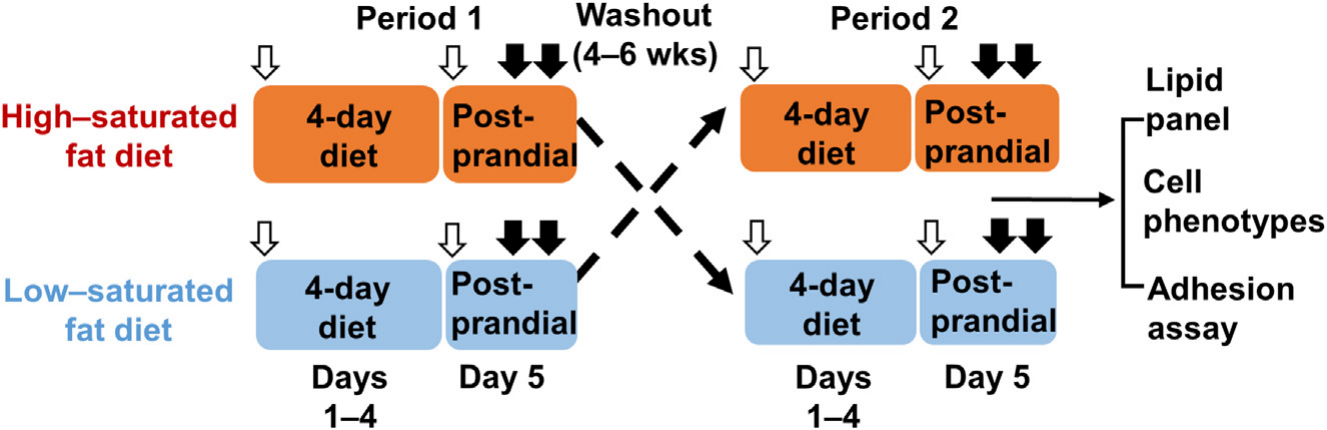
Study Design In this randomized crossover study, 19 patients with hypertriglyceridemia and metabolic syndrome received a high–saturated fat diet and a low–saturated fat diet in a randomized order separated by a 4- to 6-week washout period. Each diet period included 2 study phases: a 4-day diet phase (days 1-4) and a postprandial phase (day 5). Blood was drawn on day 1 after an overnight fast (before diet intervention) and on day 5 fasting (0 hour) and 4 and 6 hours after a test meal, which contained ∼900 kcal and was similar in composition to the respective study diet. **White arrows** indicate fasting blood draws; **closed arrows** indicate postprandial blood draws; **dashed lines** indicate washout period; and the **solid line** indicates subsequent analyses.

Each participant received HSFD and LSFD (Table 2) in a randomized order separated by a 4- to 6-week washout period (Figure 1). Each diet period included 2 study phases: a 4-day diet phase (days 1-4) and a postprandial phase (day 5). Blood was drawn on day 1 after an overnight fast (before diet intervention) and on day 5 fasting (0 hour) and 4 and 6 hours after a test meal, which contained ∼900 kcal and was similar in composition to the respective study diet. An aliquot of blood samples was sent to a local Quest Diagnostics laboratory for complete blood count analysis, and the remainder of the blood samples were processed immediately to analyze monocyte phenotypes and isolate plasma samples; these samples were stored at –80°C in small aliquots and used later for biochemical assays.

**Table 2:** Diet Intake and Composition

	Days 1-4 (Diet Phase)		Day 5 (Test Meal)	
	HSFD	LSFD	HSFD	LSFD
Calorie intake, kcal^a^	2,333.04 ± 148.59	2,118.85 ± 75.49	931.8 ± 14.65	826.27 ± 38.94^b^
Total fat, g^a^	137.63 ± 9.63	63.82 ± 2.5^c^	59.13 ± 1.56	22.25 ± 1.77^c^
SFA, g^a^	60 ± 2.41	12.47 ± 0.46^c^	35.6 ± 0.62	3.66 ± 0.39^c^
MUFA, g^a^	35 ± 3.32	29.04 ± 1.35	9.85 ± 1.15	8.81 ± 0.84
PUFA, g^a^	19.63 ± 4.54	15.83 ± 0.59	3.56 ± 0.31	5.35 ± 0.48^d^
Total carbohydrate, g^a^	189.18 ± 14.28	251.36 ± 9.62^d^	53.72 ± 1.37	102.65 ± 5.63^c^
Total protein, g^a^	88.59 ± 2.91	148.06 ± 4.91^c^	42.68 ± 0.96	60.88 ± 2.4^c^
Animal protein, g^a^	49.88 ± 1.84	100.46 ± 3.49^c^	20.12 ± 1.8	36.73 ± 1.9^c^
Vegetable protein, g^a^	16.98 ± 1.26	40.69 ± 1.63^c^	1.08 ± 0.59	12.6 ± 0.89^c^
Cholesterol, mg^a^	622.45 ± 30.44	197.73 ± 5.67^c^	406.84 ± 35.39	52.41 ± 1.33^c^
Calories from fat, %	52.34 ± 0.4	25.99 ± 0.39^c^	56.96 ± 0.85	23.11 ± 1.4^c^
SFA	23.51 ± 0.63	5.14 ± 0.16^c^	34.36 ± 0.34	3.79 ± 0.34^c^
MUFA	13.03 ± 0.3	11.67 ± 0.23^d^	9.39 ± 0.99	8.99 ± 0.71
PUFA	6.76 ± 0.69	6.45 ± 0.08	3.41 ± 0.27	5.5 ± 0.38^c^
Calories from carbohydrate, %	31.87 ± 0.54	45.86 ± 0.4^c^	23.08 ± 0.64	46.77 ± 1.35^c^
Calories from protein, %	15.72 ± 0.45	28.26 ± 0.26^c^	18.87 ± 0.29	30.61 ± 0.75^c^

Values are mean ± SEM and were compared by using paired Student’s *t*-tests. *P* values are for comparisons with corresponding phase for HSFD high–saturated fat diet (HSFD).

LSFD = low–saturated fat diet; MUFA = monounsaturated fatty acids; PUFA = polyunsaturated fatty acids; SFA = saturated fatty acids.

a Daily intake on days 1 to 4; test meal intake on day 5.

b *P* < 0.05.

c *P* < 0.001.

d *P* < 0.01.

The diets were developed by a dietitian at the Children’s Nutrition Research Center, Baylor College of Medicine, where food was prepared and provided to the participants on day 1 for the 4-day diet phase. The test meal was provided to the participants on day 5, and the postprandial study was conducted at the Center for Cardiometabolic Disease Prevention, Baylor College of Medicine, where all the blood samples were collected. During the diet periods, the participants were instructed to eat only the provided food and not other food or calorie-containing drinks, including alcohol, and to maintain their usual physical activity. Dietary intake and the compliance of each participant were monitored by the dietitian. During the washout period, the participants were advised to eat their usual diet.

### Biochemical Assays

Total cholesterol, triglyceride, HDL-C, and glucose were measured in ethylenediaminetetraacetic acid (EDTA) plasma using enzymatic methods on a Beckman AU480 automated chemistry analyzer (Beckman Coulter). Low-density lipoprotein cholesterol (LDL-C) was calculated according to the Friedewald formula. A homogeneous assay method was used for the direct measurement of small dense LDL-C in plasma (sd-LDL-EX “Seiken,” Denka Seiken) on a Beckman AU480 automated chemistry analyzer. Large buoyant LDL-C was estimated by subtracting the small dense LDL-C concentration from the LDL-C concentration. Plasma apolipoprotein CIII (apoCIII) (APOC3, Kamiya Biomedical Company) and high-sensitivity C-reactive protein (hsCRP) (Sekisui Diagnostics) were measured by using an immunonephelometric assay with a Beckman AU480 automated chemistry analyzer. Remnant-like lipoprotein particle cholesterol (RLP-C) and LDL triglyceride (LDL-TG) were determined by using fully automated detergent-based homogeneous methods (Denka Seiken).^26^ Insulin levels were determined by electrochemiluminescence immunoassay on a Roche Cobas e411 immunoanalyzer (Roche Diagnostics). Homeostatic model assessment for insulin resistance was calculated as: fasting serum insulin (microunits per milliliter) × fasting plasma glucose (millimoles per liter)/22.5.

Fatty acids and glycoprotein acetylation (GlycA) were measured in EDTA plasma samples by a high-throughput metabolomics platform (biomarker quantification version 2020, Nightingale Health Ltd).^27^

### Flow Cytometric Analysis of Monocyte Lipid Accumulation and Phenotypes

Monocyte phenotypes were analyzed by using flow cytometry as described previously.^16^^,^^18^^,^^28^ Briefly, for cell surface marker analysis, 100 μL of EDTA blood was incubated with a mixture of antibodies (Supplemental Table 1) in 2 different protocols (Protocols 1 and 2) (Supplemental Table 2) at 4°C for 30 minutes and then fixed with BD lysing solution (BD Biosciences), which also lysed red blood cells. Leukocytes were pelleted and washed with phosphate-buffered solution (PBS) supplemented with 1% bovine serum albumin and suspended in 2% paraformaldehyde in PBS for detection by using a Gallios cytometer (Beckman Coulter).

For Nile Red staining of lipids, blood was first incubated with a mixture of antibodies for cell surface markers (Protocol 3) (Supplemental Table 2). After red blood cells were lysed and the samples were fixed with BD lysing solution, cells were stained with Nile Red (Sigma-Aldrich) at 0.1 μM in PBS for 20 minutes. The samples were washed with PBS 2 more times and finally resuspended in 2% paraformaldehyde in PBS for detection by flow cytometry.

For oxidized low-density lipoprotein (oxLDL) uptake assay, 100 μL of blood was mixed with an equal volume of complete RPMI 1640 medium and incubated with 120 μg (protein)/mL DiI-labeled human oxLDL (Kalen Biomedical) at 37°C for 1 hour. Samples were then subjected to cell surface marker staining (Protocol 4) (Supplemental Table 2) as described earlier and suspended in 2% paraformaldehyde in PBS for detection by flow cytometry.

For data analysis of monocytes and subsets, total leukocytes were first gated based on forward scatter and side scatter (SSC). Monocytes were identified as CD14^+^ leukocytes, which were further classified into 3 subsets—classical (cM), intermediate (iM), and nonclassical (nM) monocytes—based on CD14 and CD16.^18^ Lipid accumulation in monocytes and subsets was assessed by SSC values (representing cell granularity) and Nile Red staining.^21^, ^22^, ^23^ The expression level of adhesion molecules (CD11a, CD11c, CD62L, CD81, and very late activation antigen-4 [VLA4]), chemokine receptors (CCR2, CCR5, and CX3CR1), scavenger receptors (CD36, scavenger receptor type A, LDL receptor–related protein 1), and human leukocyte antigen-DR isotype on monocytes/subsets was presented as mean fluorescence intensity of staining for the specific molecules. Monocyte uptake of oxLDL was estimated according to DiI mean fluorescence intensity levels of monocytes/subsets after coincubation with DiI-labeled oxLDL.^23^

### On-Chip Adhesion Assay

Monocyte adhesion assay was performed by using whole blood as previously reported.^23^^,^^29^^,^^30^ Briefly, coverslips that were coated with recombinant human vascular cell adhesion molecule-1/Fc chimera (R&D Systems) were assembled with a 4-channel polydimethylsiloxane device and kept at 37°C. Heparin-anticoagulated blood (100 μL) was stained with phycoerythrin–anti-CD16 and Alexa Fluor 488–anti-CD14 antibodies at room temperature for 20 minutes. After 1:3 dilution in PBS with calcium and magnesium, 60 μL of diluted blood was introduced into the polydimethylsiloxane device at a flow rate that produced a shear stress of 2 dynes/cm^2^ at the fluid–glass interface and was perfused for 5 minutes, followed by fixation, and mounting in permanent 4′,6-diamidino-2-phenylindole medium. The number of adherent monocytes (subsets) was counted by 3 individuals blinded to diet intervention and normalized by the total infused number of each monocyte subset.

### Statistical Methods

The Shapiro-Wilk test was used to determine the data normality. Data with normal distribution are presented as mean ± SEM; otherwise, data are presented as median with 25th and 75th percentiles (Q1, Q3). All data were plotted by using Prism 8 or higher (GraphPad Software). Statistical analyses were performed with Prism 8 or higher or R (Version 4.2.2, R Foundation for Statistical Computing). Data distributions and variances were evaluated by using Bartlett’s test, and variables displaying heterogeneous variance were log transformed for statistical analyses and transformed back for presentation. Diet intake and composition were compared between HSFD and LSFD by using paired Student’s *t*-tests. Linear mixed-effects models with a crossover design and repeated measures were performed (package “lme4”; R Foundation for Statistical Computing) to analyze factors of diet interventions over time (within-subject factor of time and diet; time × diet interaction) in the 4-day diet phase (from day 1 fasting to day 5 fasting) and the postprandial phase (including fasting and 4 and 6 hours postprandially on day 5). In this repeated-measure crossover design, we defined a compound symmetrical variance–covariance structure in the model constructed by using the lme4 package. Post hoc tests of diets within each time factor were conducted with multiple pairwise comparisons using estimated marginal means (package “emmeans;” R Foundation for Statistical Computing), with *P* values adjusted by using the Bonferroni method. Variables of age, sex, body weight, and order of diets (LSFD after HSFD and HSFD after LSFD) were added to the linear mixed-effects model to adjust for confounding effects. No significant crossover effect from order of diets was found. Spearman correlations (*r*) were calculated between monocyte phenotype markers and lipid variables. *P* values ≤0.05 were considered statistically significant unless otherwise specified.

### Study Approval

The study protocol (H-21418) was approved by the Institutional Review Board of Baylor College of Medicine. All participants provided written informed consent before participation.

## Results

### Baseline Characteristics of Participants and Diet Intake

Nineteen adults (10 men and 9 women) with hypertriglyceridemia and metabolic syndrome were included. The baseline characteristics of participants are shown in Table 1. The mean ± SEM age of the study cohort was 53.9 ± 3.20 years, mean body mass index was 37.2 ± 2.57 kg/m^2^, mean fasting triglyceride level was 232.1 ± 26.4 mg/dL, and mean HDL-C level was 42.4 ± 1.71 mg/dL. The median (Q1, Q3) hsCRP level was 2.6 (0.86, 10.3) mg/L.

To examine the effects of LSFD vs HSFD, each participant received HSFD and LSFD (Table 2) in a randomized order separated by a 4- to 6-week washout period (Figure 1). Each diet period included 2 study phases: a 4-day diet phase (days 1-4) and a postprandial phase (day 5). Daily calorie intake was not significantly different between LSFD and HSFD in the 4-day diet phase, but the calorie intake of the test meal on day 5 was slightly but significantly lower for LSFD than for HSFD. As designed, fat intake was significantly lower, and protein and carbohydrate intake was higher with LSFD than with HSFD. Of the fat intake, saturated fatty acid intake was significantly lower with LSFD than with HSFD, whereas monounsaturated fatty acid and polyunsaturated fatty acid intake showed no or minor differences. With HSFD, ∼52% of calories were from fat and ∼24% of calories were from saturated fat in the 4-day diet phase, and ∼57% of calories were from fat and ∼34% of calories were from saturated fat in the test meal; with LSFD, ∼26% of calories were from fat and ∼5% of calories were from saturated fat in the 4-day diet phase, and ∼23% of calories were from fat and ∼3.8% of calories were from saturated fat in the test meal. Cholesterol intake was lower with LSFD than with HSFD.

### Changes in Plasma Levels of Lipids and Inflammatory Markers With LSFD or HSFD

**Figure 2:**
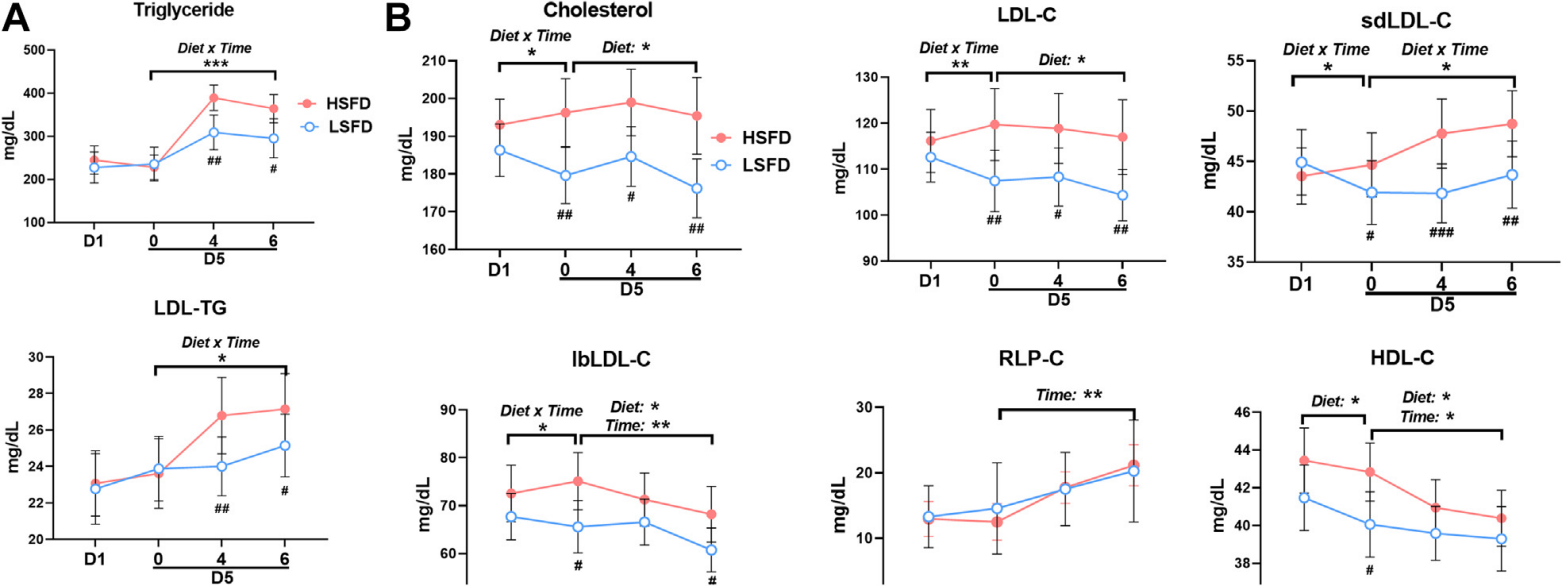

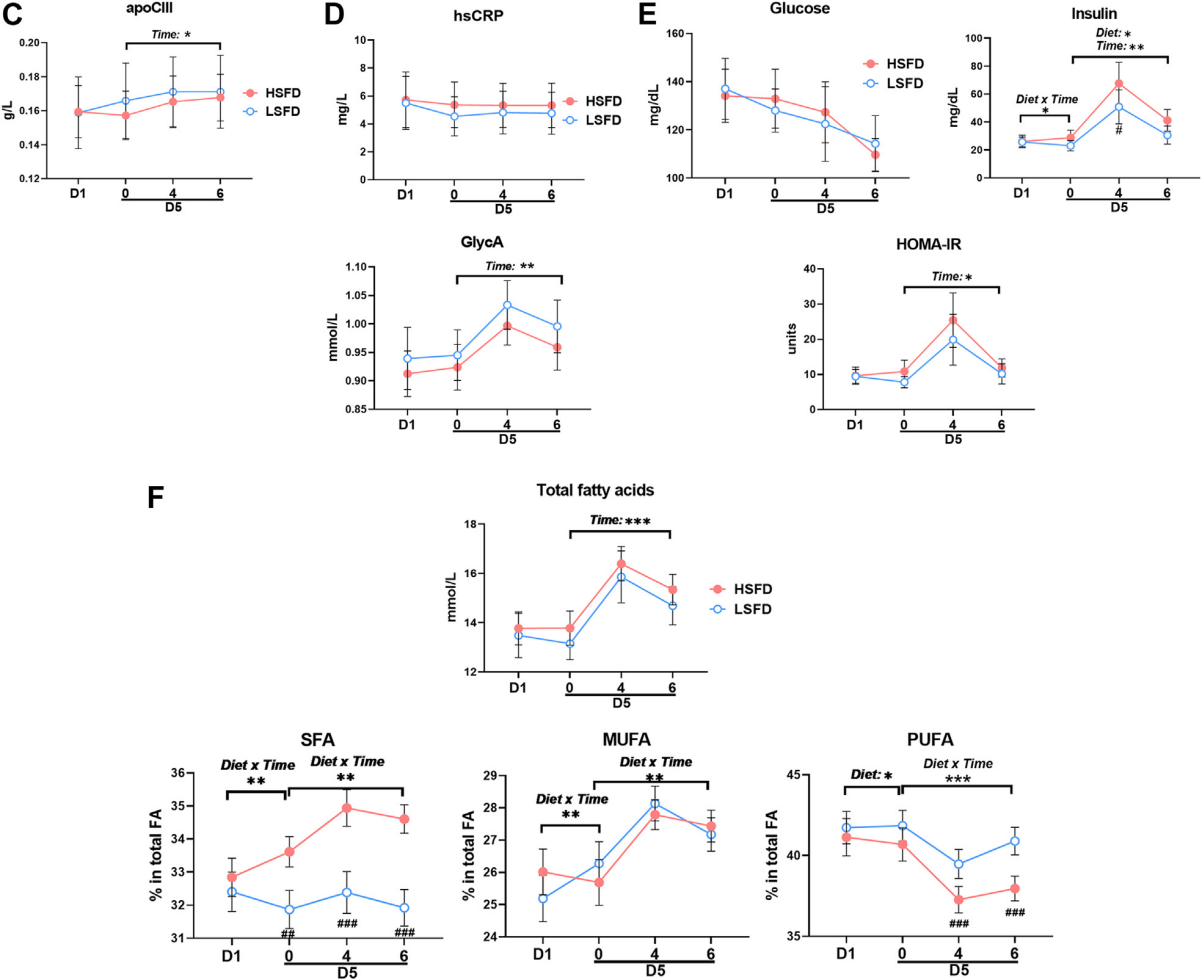
Biochemical Profiles **(A)** Plasma levels of triglyceride and low-density lipoprotein triglyceride (LDL-TG). **(B)** Plasma levels of total cholesterol; LDL cholesterol (LDL-C), including small dense LDL-C (sdLDL-C) and large buoyant LDL-C (lbLDL-C); remnant-like particle cholesterol (RLP-C); and high-density lipoprotein cholesterol (HDL-C). **(C)** Plasma levels of apolipoprotein CIII (apoCIII). **(D)** Plasma levels of high-sensitivity C-reactive protein (hsCRP) and glycoprotein acetylation (GlycA). **(E)** Plasma levels of glucose, insulin, and homeostatic model assessment for insulin resistance (HOMA-IR). **(F)** Plasma levels of total fatty acids and percentages of saturated fatty acids (SFA), monounsaturated fatty acids (MUFA), and polyunsaturated fatty acids (PUFA) in total fatty acids. Values are mean ± SEM. ∗*P* < 0.05, ∗∗*P* < 0.01, ∗∗∗*P* < 0.001 for main effects; ^#^*P* < 0.05, ^##^*P* < 0.01, ^###^*P* < 0.001 post hoc test for pairwise comparisons between diets at indicated time points (day 1 [D1] fasting; day 5 [D5] fasting [0] and 4 hours [4] and 6 hours [6] after a test meal). HSFD = high–saturated fat diet; LSFD = low–saturated fat diet.

To investigate the effects of the diets on plasma lipid levels, we first profiled the plasma lipid panel. Plasma triglyceride levels did not change in the 4-day diet phase with either diet and did not differ between the 2 diets (Figure 2A). As expected, triglyceride levels were increased postprandially, and a significant interaction of diet by time was observed in the postprandial phase. Compared with HSFD, LSFD induced significantly lower postprandial triglyceride levels at 4 and 6 hours. In addition to the enrichment of triglyceride in triglyceride-rich lipoproteins (ie, very-low-density lipoproteins, chylomicrons, and their remnants), triglyceride in LDL (ie, LDL-TG) is associated with hypertriglyceridemia and an increased risk of ASCVD.^9^^,^^31^ Similar to changes in total triglyceride, changes in LDL-TG also exhibited a significant diet × time interaction in the postprandial, but not the 4-day diet, phase. These data indicate that after 4 days, neither LSFD nor HSFD altered fasting plasma levels of triglyceride or LDL-TG. However, compared with HSFD, LSFD induced a significantly lower postprandial response in total triglyceride and LDL-TG.

Changes in plasma levels of total cholesterol and LDL-C exhibited a significant diet × time interaction in the 4-day diet phase and a diet effect in the postprandial phase (Figure 2B). In the 4-day diet phase, HSFD increased while LSFD reduced fasting total cholesterol and LDL-C levels, consistent with previous studies.^14^ In the postprandial phase, LDL-C levels decreased or tended to decrease, primarily because of reductions in large buoyant LDL-C, with both diets. Compared with HSFD, LSFD induced lower total cholesterol and lower LDL-C levels fasting and postprandially. Changes in small dense LDL-C levels had significant diet × time interactions in both the 4-day diet phase and the postprandial phase, and small dense LDL-C levels were lower in the fasting state and 4 and 6 hours postprandially with LSFD vs HSFD; large buoyant LDL-C levels exhibited significant diet × time interactions in the 4-day diet phase, and they were lower fasting and 6 hours postprandially with LSFD than with HSFD. The lower postprandial small dense LDL-C levels, along with lower postprandial total triglyceride and LDL-TG levels, with LSFD vs HSFD were consistent with previous reports.^32^^,^^33^ These findings showed that more severe and prolonged elevations in postprandial triglycerides (as in our studies with HSFD) lead to increased transfer of triglycerides from chylomicrons and very-low-density lipoprotein to LDL in exchange for cholesteryl esters, resulting in an increase in LDL-TG and generation of small dense LDL particles after interaction with hepatic lipase. It would also be important in future studies to examine the effects of different diets on particle numbers of these lipoproteins. Levels of RLP-C exhibited postprandial increases but did not differ between the diets (Figure 2B). The similar levels of RLP-C with HSFD and LSFD, despite lower postprandial triglyceride levels in LSFD, were likely because most of the triglycerides in the early postprandial state are in chylomicrons. The RLP-C assay that we used was developed for measurement in overnight fasting samples and measured the cholesterol in very-low-density lipoprotein remnants, not in chylomicron remnants.^34^ HDL-C levels decreased over time, with time and diet effects, and were lower in the fasting state with LSFD vs HSFD. Levels of apoCIII and GlycA exhibited postprandial increases but were not different between the diets (Figures 2C and 2D). In contrast, hsCRP did not exhibit any diet or time effects (Figure 2D).

Glucose levels tended to decrease over time in both the 4-day diet and postprandial phases, with no differences between the diets (Figure 2E). Changes in plasma insulin levels displayed diet × time interactions in the 4-day diet phase and diet and time effects in the postprandial phase. Insulin resistance showed significant time effects in the postprandial phase.

### Changes in Plasma Levels of Fatty Acids With LSFD or HSFD

Using nuclear magnetic resonance, we examined changes in plasma levels of fatty acids with LSFD or HSFD (Supplemental Table 3). Total fatty acid levels did not change in the 4-day diet phase but were increased in the postprandial phase, with no differences between the 2 diets (Figure 2F, Supplemental Table 3). However, plasma fatty acid compositions differed vastly between the 2 diets. The percentages of saturated fatty acids in total fatty acids showed significant diet × time interactions in both the 4-day diet and postprandial phases, and they were significantly lower in the fasting state and 4 and 6 hours postprandially with LSFD vs HSFD. In contrast, the percentages of polyunsaturated fatty acids were higher at 4 and 6 hours postprandially with LSFD than with HSFD, with a significant diet × time interaction in the postprandial phase.

### LSFD Compared With HSFD Reduced Monocyte Lipid Accumulation

**Figure 3:**
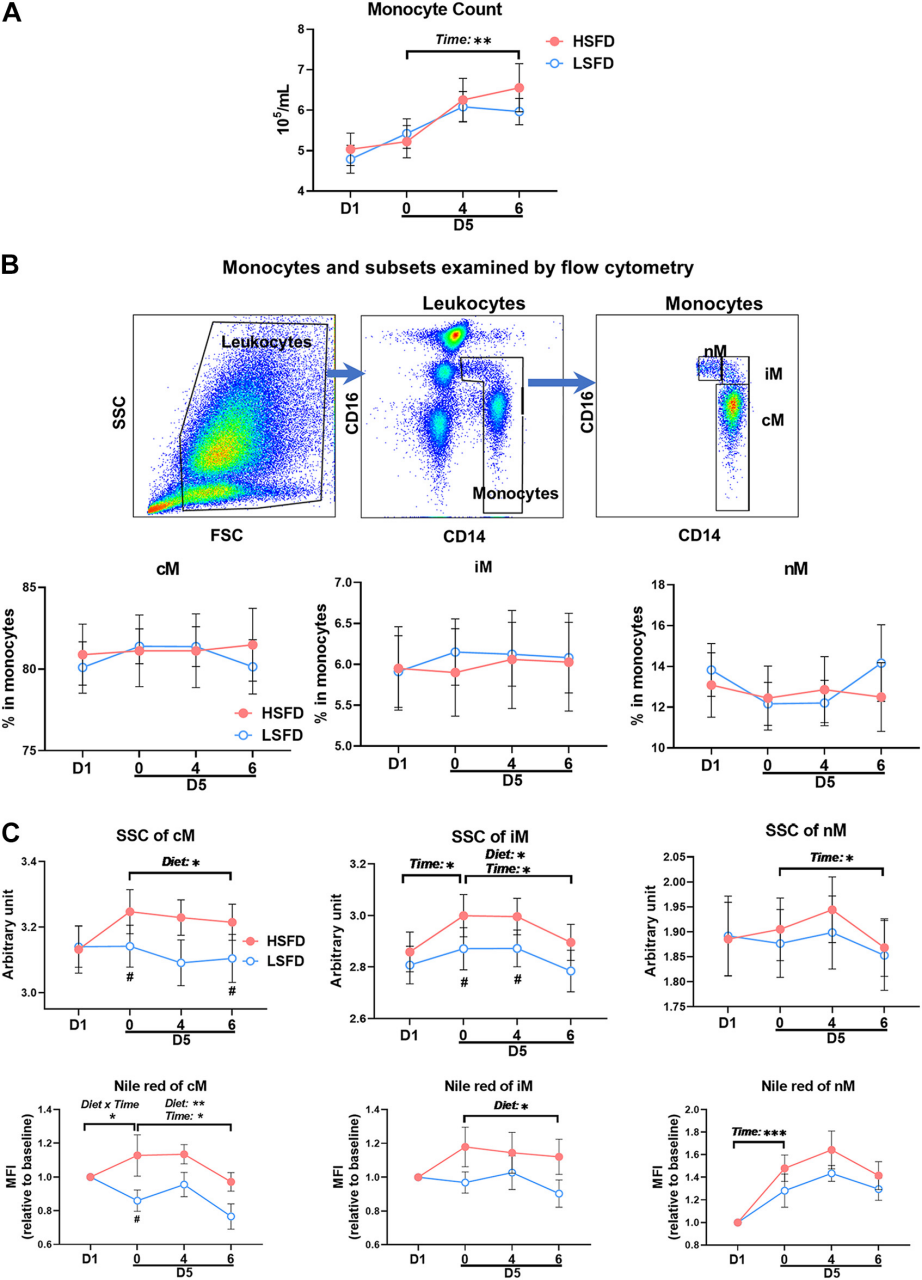
Changes in Monocyte Counts, Subset Frequencies, and Lipid Accumulation **(A)** Total monocyte counts. **(B)** Representative flow cytometric examples and frequencies of monocyte subsets: CD14^++^/CD16^–^ classical (cM), CD14^+^/CD16^+^ intermediate (iM), and CD14^dim^/CD16^+^ nonclassical (nM) monocytes. **(C)** Side scatter (SSC), representing cell granularity/lipid droplets, and Nile Red staining for lipids (indicated by relative mean fluorescence intensity [MFI] of Nile Red staining) of monocyte subsets. Values are mean ± SEM. ∗*P* ≤ 0.05, ∗∗*P* < 0.01, ∗∗∗*P* < 0.001 for main effects; ^#^*P* < 0.05 post hoc test for pairwise comparisons between diets at indicated time points. D5 = day 5; FSC = forward scatter; other abbreviations as in Figure 2.

We next focused on diet effects on monocyte phenotypes. Total white blood cell counts increased in both the 4-day diet and postprandial phases and had a diet × time interaction in the postprandial phase (Supplemental Figure 1). Among white blood cells, monocyte, neutrophil, and lymphocyte counts all tended to increase in the 4-day diet phase and increased in the postprandial phase (Figure 3A, Supplemental Figure 1). In contrast, no diet or time effects were observed in eosinophil, basophil, red blood cell, and platelet counts.

Based on CD14 and CD16, human monocytes are classified into CD14^++^/CD16^–^ cM, CD14^+^/CD16^+^ iM, and CD14^dim^/CD16^+^ nM (Figure 3B), which are also different in numerous intracellular and cell surface markers and the role in inflammation.^35^ Although CD16^+^ iM/nM account for small portions of total monocytes, studies have shown positive correlations of CD16^+^ iM/nM with ASCVD or progression of carotid thickness.^36^, ^37^, ^38^ We observed that despite the changes in total monocyte counts, the percentage of each monocyte subset in total monocytes did not change, with no diet or time effects.

Lipid accumulation in monocytes, leading to foamy monocyte formation, occurs in humans and mice with hyperlipidemia and may contribute to atherosclerosis development.^16^, ^17^, ^18^, ^19^, ^20^, ^21^, ^22^^,^^39^ We therefore examined lipid accumulation in monocytes as indicated by changes in SSC (representing cell granularity and lipid droplets in monocytes^16^^,^^18^^,^^21^^,^^22^) and Nile Red staining^22^ from flow cytometric analysis. As shown in Figure 3C, diet effects were observed in lipid accumulation of cM and iM in the postprandial phase. Compared with HSFD, LSFD induced significantly lower SSC, indicating less lipid accumulation, in cM fasting and 4 and 6 hours postprandially and in iM fasting and 4 hours postprandially. Nile Red staining confirmed less lipid accumulation in cM and iM with LSFD than with HSFD.

### Changes in Monocyte Adhesion Molecules, Chemokine Receptors, and Adhesion to Vascular Cell Adhesion Molecule-1

Lipid accumulation can change monocyte phenotypes, leading to alterations in monocyte functions.^17^^,^^18^^,^^21^^,^^22^^,^^39^ Because of the changes in lipid accumulation, we next analyzed diet effects on monocyte phenotypes, beginning with monocyte levels of adhesion molecules and chemokine receptors, which play pivotal roles in monocyte transendothelial migration and therefore contribute to inflammation, including atherosclerosis.

**Figure 4:**
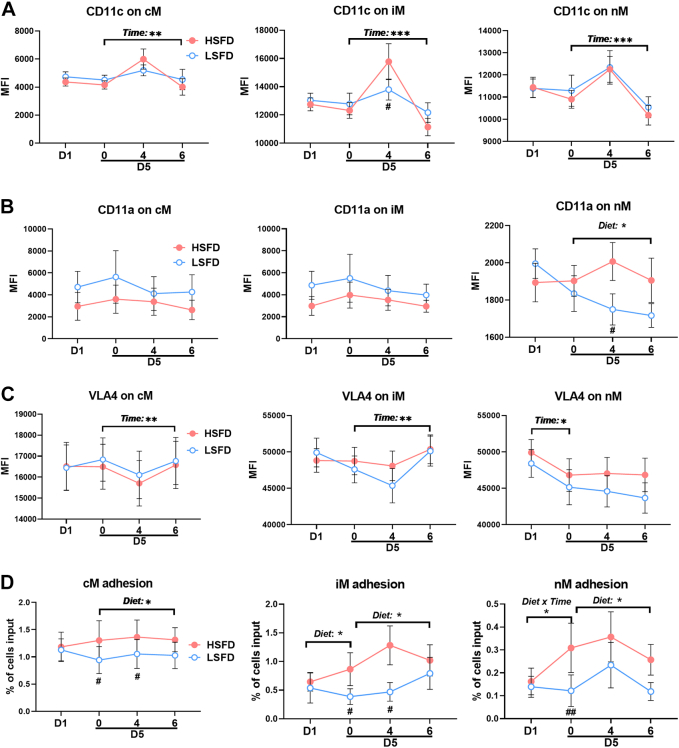
Adhesion Molecules and Adhesion Capacity of Monocytes Expression levels, indicated by MFI, of adhesion molecules, including CD11c **(A)**, CD11a **(B)**, and very late activation antigen-4 (VLA4) **(C)**, on monocyte subsets examined by flow cytometry. **(D)** Firm adhesion of monocyte subsets on vascular cell adhesion molecule-1 examined by the ex vivo micro-flow adhesion assay. Values are mean ± SEM. ∗*P* ≤ 0.05, ∗∗*P* < 0.01, ∗∗∗*P* < 0.001 for main effects; ^#^*P* < 0.05, ^##^*P* < 0.01 post hoc test for pairwise comparisons between diets at indicated time points. Abbreviations as in Figures 2 and 3.

CD11c, also known as αX integrin, mediates monocyte adhesion by enhancing VLA4 affinity for vascular cell adhesion molecule-1, an adhesion molecule expressed on activated endothelial cells.^17^^,^^29^ At baseline (before diet interventions), CD11c levels were higher on iM and nM than on cM, consistent with our previous reports.^16^^,^^18^ Neither a time nor diet effect was observed in CD11c levels on any monocyte subsets in the 4-day diet phase, but a time effect was observed in CD11c on all subsets in the postprandial phase (Figure 4A). Compared with HSFD, LSFD induced lower CD11c levels on iM at 4 hours postprandially. CD11a, also known as integrin αL and belonging to the same integrin family as CD11c, participates in transendothelial migration of monocytes, nM in particular,^40^ and other leukocytes.^41^ We observed no diet or time effects in cM and iM CD11a levels in either phase; however, a diet effect in nM CD11a levels was observed in the postprandial phase, with lower nM CD11a at 4 hours with LSFD than with HSFD (Figure 4B). Time effects were observed in cM and iM VLA4 levels in the postprandial phase and in nM VLA4 in the 4-day diet phase, but no diet effects were observed in VLA4 levels on any monocyte subsets (Figure 4C).

CD81 is an integrin-associated tetraspanin that is expressed on leukocytes, including monocytes, and also on endothelial cells; it mediates leukocyte transendothelial migration.^42^ We observed a time effect in iM and nM CD81 levels in the postprandial phase and a trend toward lower CD81 levels on iM and nM with LSFD vs HSFD (Supplemental Figure 2).

CD62L shedding mediated by enzymatic cleavage occurs during monocyte activation. We observed a time effect in cM CD62L levels in the postprandial phase and a trend toward higher cM CD62L levels, indicating less CD62L shedding and lower activation status, with LSFD vs HSFD (Supplemental Figure 2).

Importantly, with the aforementioned changes in monocyte adhesion molecules, we observed diet effects or diet × time interactions in monocyte adhesion on vascular cell adhesion molecule-1 examined by the micro-flow adhesion assay (Figure 4D). Compared with HSFD, LSFD decreased firm adhesion of cM and iM on vascular cell adhesion molecule-1 in the fasting state and 4 hours postprandially and decreased or tended to decrease nM adhesion fasting and 6 hours postprandially.

Taken together, compared with HSFD, LSFD induced lower levels of several adhesion molecules on monocytes and reduced monocyte firm adhesion on vascular cell adhesion molecule-1.

Analyses of chemokine receptors showed that at baseline, CCR2, the receptor for CCL2, was higher, but CX3CR1, the receptor for CX3CL1, was lower on cM than on nM, with iM expressing intermediate CCR2 and CX3CR1 levels (Supplemental Figure 3), consistent with previous reports.^43^^,^^44^ The changes in cM CCR2 levels tended to have a diet × time interaction in the 4-day diet phase, with lower cM CCR2 levels while fasting on day 5 of LSFD vs HSFD, whereas nM CCR2 levels tended to have a diet × time interaction in the postprandial phase and tended to be lower postprandially with LSFD vs HSFD. We observed a time effect, but no diet effect, in monocyte CX3CR1 levels in the postprandial phase. Neither diet nor time effects were observed in CCR5 and human leukocyte antigen-DR levels on any monocyte subsets.

### Changes in Monocyte Scavenger Receptors and Uptake of oxLDL

Monocyte/macrophage uptake of modified LDL, such as oxLDL, is a key step in atherosclerotic foam cell formation and is mainly mediated by scavenger receptors.^22^ We therefore analyzed whether LSFD and HSFD differentially affected monocyte expression of scavenger receptors, including CD36 and scavenger receptor A, and monocyte uptake of oxLDL ex vivo.

**Figure 5:**
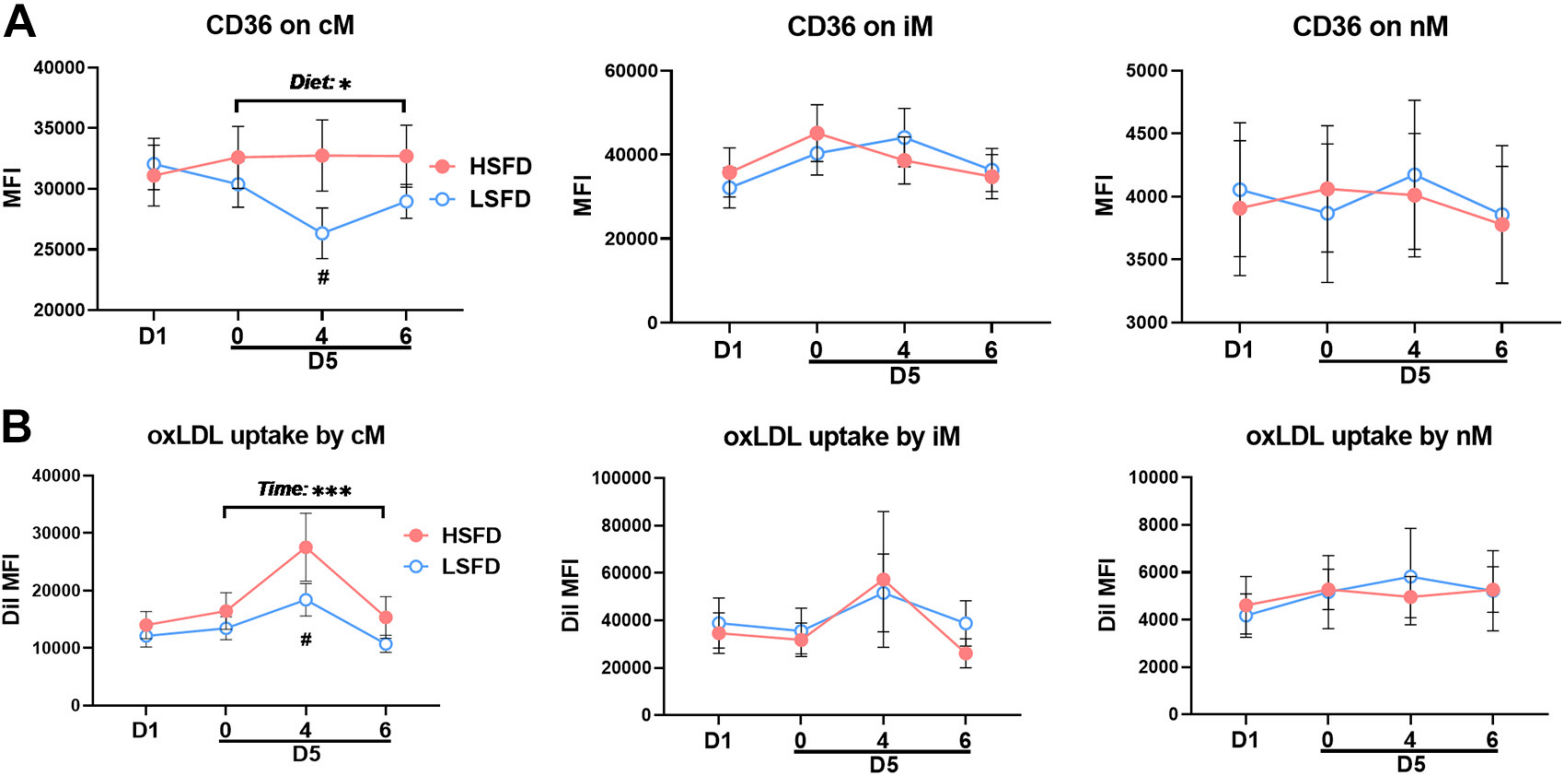
Monocyte CD36 Levels and oxLDL Uptake Ex Vivo **(A)** Expression levels of CD36, indicated by MFI, on monocyte subsets examined by flow cytometry. **(B)** Oxidized low-density lipoprotein (oxLDL) uptake, presented as DiI (oxLDL) MFI, by monocyte subsets examined by using flow cytometry after coincubation of DiI-labeled oxLDL with monocytes ex vivo for 1 hour. Values are mean ± SEM. ∗*P* < 0.05, ∗∗∗*P* < 0.001 for main effects; ^#^*P* < 0.05 post hoc test for pairwise comparisons between diets at indicated time points. Abbreviations as in Figures 2 and 3.

At baseline, CD36 levels were higher on cM and iM than on nM (Figure 5A), consistent with previous reports.^16^^,^^44^ In the postprandial phase, a diet effect was observed in cM CD36 levels, which were significantly lower at 4 hours with LSFD than with HSFD. In contrast, scavenger receptor A levels were higher on nM and iM than on cM at baseline but were generally low on all monocyte subsets and did not show any diet effects (Supplemental Figure 4). No diet or time effects were observed in monocyte levels of LDL receptor–related protein 1. After incubation with DiI-labeled oxLDL ex vivo, all monocyte subsets displayed oxLDL uptake, with the greatest uptake by iM, as indicated by DiI mean fluorescence intensity levels (Figure 5B). A time effect was observed in cM uptake of oxLDL in the postprandial phase. Compared with HSFD, LSFD induced less oxLDL uptake by cM at 4 hours in the postprandial phase.

### Correlations of Monocyte Phenotypes With Plasma Levels of Lipids

**Figure 6:**
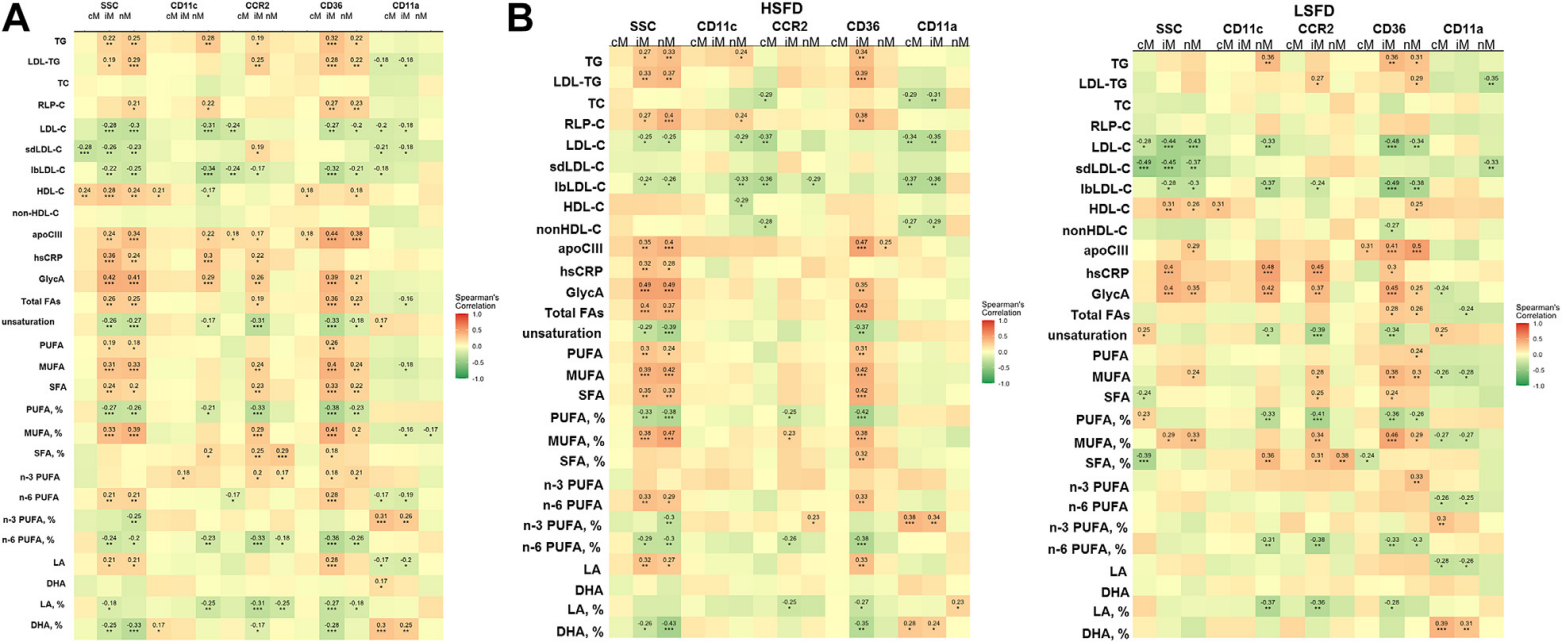
Correlations Between Monocyte Markers and Plasma Levels of Lipids **(A)** Data from all time points and both diets combined. **(B)** Data according to diet but combined from all time points for each diet. The x-axis indicates monocyte markers, and the y-axis indicates lipid variables. DHA = docosahexaenoic acid; FA = fatty acid; HDL-C = high-density lipoprotein cholesterol; LA = linoleic acid; TC = total cholesterol; TG = triglyceride; other abbreviations as in Figures 2 and 3.

In dyslipidemia, changes in plasma levels of lipids may be the main cause for monocyte lipid accumulation and phenotypic changes. In our exploratory approaches to determine the specific lipids contributing to monocyte lipid accumulation and phenotypic changes, we performed simple correlation analyses between monocyte lipid accumulation/phenotypes and plasma levels of lipids (Figure 6).

Analyses of all data generated from both diets showed that monocyte lipid accumulation (indicated by SSC) and phenotypes, including surface levels of CD11c, CCR2, and CD36, were associated with several lipid variables. For example, monocyte SSC values and levels of CD11c, CCR2, and CD36, on iM and/or nM in particular, but not on cM, were positively associated with plasma levels of triglycerides, LDL-TG, RLP-C, apoCIII, and total fatty acids, as well as the inflammatory markers hsCRP and GlycA. Of the fatty acids, percentages of saturated fatty acids and monounsaturated fatty acids largely showed positive correlation with the monocyte markers (Figure 6A). Further analyses of data stratified according to diet indicated that most of the associations between lipid variables and monocyte markers were derived from HSFD, whereas the correlations between plasma levels of inflammatory markers (hsCRP and GlycA) and monocyte markers were observed in both HSFD and LSFD (Figure 6B). Of note, we observed that plasma levels of total cholesterol were not associated with most of the monocyte markers, whereas plasma levels of LDL-C, particularly large buoyant LDL-C, were negatively associated with most of the monocyte markers in these subjects with hypertriglyceridemia with both HSFD and LSFD.

## Discussion

Diet interventions are an important regimen for ASCVD prevention. Although Western diets high in saturated fat and cholesterol have been associated with ASCVD risk, diets high in fat and protein but low in carbohydrates have recently become popular for reducing body weight in patients with metabolic syndrome.^14^^,^^25^ In the current study, we showed that in patients with hypertriglyceridemia and metabolic syndrome, short-term intervention with LSFD or HSFD was associated with different monocyte responses. Compared with HSFD, LSFD reduced fasting and postprandial intracellular lipid accumulation in cM and iM. Consistently, following LSFD vs HSFD, surface levels of molecules participating in monocyte adhesion and migration, including CD11c, CD11a, and CCR2, were lower or tended to be lower on monocytes, with reduced monocyte adhesion to vascular cell adhesion molecule-1. Compared with HSFD, LSFD also reduced cM CD36 levels and cM uptake of oxLDL ex vivo. Furthermore, monocyte lipid accumulation and surface levels of CD11c, CCR2, and CD36 were mostly positively associated with plasma levels of triglyceride, LDL-TG, RLP-C, apoCIII, fatty acids, and inflammatory markers but not with total cholesterol. Given the pivotal role of monocytes in atherosclerosis, the differential responses of monocytes to LSFD and HSFD may contribute to the differences in ASCVD risk associated with diets that differ in fat content and composition.^14^ Of note, considering no or minor differences in unsaturated fat between LSFD and HSFD, our data suggest potential adverse effects of high intake of saturated fat on ASCVD risk in subjects with hypertriglyceridemia and metabolic syndrome.

It is important to emphasize that the subjects in our studies had metabolic syndrome and hypertriglyceridemia, which, in addition to hypercholesterolemia, is recognized as an important causal risk factor for ASCVD.^1^, ^2^, ^3^, ^4^, ^5^ However, the method by which hypertriglyceridemia increases ASCVD risk remains incompletely understood. Recent studies indicate that compared with hypercholesterolemia, hypertriglyceridemia has stronger associations with inflammation, which has been critically implicated in ASCVD.^2^^,^^5^, ^6^, ^7^, ^8^, ^9^, ^10^, ^11^, ^12^, ^13^ Varbo et al^7^ reported that elevated RLP-C caused both inflammation and ASCVD, whereas elevated LDL-C caused ASCVD without inflammation. Therefore, inflammation likely plays a more important role in ASCVD that is associated with hypertriglyceridemia than in ASCVD associated with hypercholesterolemia. It warrants consideration in future studies to test whether anti-inflammatory therapies have more benefits for ASCVD prevention in subjects with hypertriglyceridemia than in individuals without hypertriglyceridemia.

It is noteworthy that our current studies showed that LSFD compared with HSFD reduced plasma levels of postprandial triglycerides and LDL-TG and fasting and postprandial total cholesterol and LDL-C, including small dense LDL-C. These findings support a potential benefit of reducing dietary intake of saturated fat for ASCVD prevention in subjects with hypertriglyceridemia and metabolic syndrome. In contrast, the recent PROMINENT (Pemafibrate to Reduce Cardiovascular Outcomes by Reducing Triglycerides in Patients with Diabetes) trial showed no benefits for ASCVD prevention in individuals with mild to moderate hypertriglyceridemia and type 2 diabetes of lowering triglyceride, RLP-C, and apoCIII levels by pemafibrate, which also raised LDL-C levels^45^ and did not change small dense LDL-C levels.^46^ It would be important in future studies to test whether alternative therapies in development, which target apoCIII and angiopoietin-related protein 3 and lower not only triglycerides but also non–HDL-C, apolipoprotein B, and small dense LDL, have benefits for ASCVD prevention in subjects with hypertriglyceridemia.

It has been recognized that triglyceride-rich lipoprotein–derived RLP can penetrate arterial walls and promote foam cell formation and is therefore atherogenic.^2^^,^^6^ It is also important to note that in addition to macrophage uptake of lipids and foam cell formation in atherosclerotic plaques, monocytes can take up lipoproteins and become lipid-laden foamy monocytes in the circulation in hypertriglyceridemia and/or hypercholesterolemia.^16^, ^17^, ^18^, ^19^, ^20^, ^21^, ^22^^,^^39^ In humans, postprandial hyperlipidemia after a single high–saturated fat meal can induce foamy monocyte formation in the circulation.^17^, ^18^, ^19^, ^20^ Lipid accumulation in monocytes alters monocyte phenotypes, leading to increased expression of activated CD11c and VLA4 that mediate enhanced adhesion to endothelial cell adhesion molecule vascular cell adhesion molecule–1.^17^^,^^22^^,^^29^ Indeed, foamy monocytes infiltrate into arterial walls, become foam cells, and contribute to the development of atherosclerosis in mouse models.^21^^,^^22^

Our current study revealed that in patients with hypertriglyceridemia and metabolic syndrome, LSFD vs HSFD induced less lipid accumulation in circulating monocytes, which was associated with several changes in monocyte phenotypes that may contribute to the beneficial effects of LSFD for ASCVD prevention. First, reduced surface levels of several adhesion molecules, with decreased monocyte (subset) adhesion to vascular cell adhesion molecule-1, after LSFD is expected to decrease monocyte infiltration into arterial walls and may therefore decelerate atherosclerosis progression. Second, reductions in monocyte CD36 expression and monocyte uptake of modified LDL with LSFD may further decrease foam cell formation and therefore may also contribute to atherosclerosis prevention. In addition, numerous studies have indicated that foam cells are mostly proinflammatory.^47^^,^^48^ We also reported increased tumor necrosis factor α and interleukin-1β levels in foamy monocytes in apolipoprotein E^–/–^ mice.^22^ However, recent reports showed that foam cells in atherosclerotic lesions did not express high levels of cytokines.^49^^,^^50^ It remains to be determined whether less lipid accumulation in monocytes with LSFD is associated with changes in monocyte cytokine levels.

Elevations in plasma lipids are likely to be the main cause for foamy monocyte formation and monocyte phenotypic changes. Our current observations that plasma lipids related to triglyceride, but not total cholesterol, were positively associated with monocyte lipid accumulation and phenotypic changes are consistent with previous reports that hypertriglyceridemia vs hypercholesterolemia correlated better with inflammation.^2^^,^^5^, ^6^, ^7^, ^8^, ^9^, ^10^, ^11^, ^12^, ^13^^,^^18^ Importantly, our study further showed that in addition to total triglyceride levels, composition of fatty acids may play an important role in monocyte lipid accumulation and phenotypic changes. In particular, the positive association of the proportion of saturated fatty acids with monocyte lipid accumulation and phenotypes is consistent with the role of saturated fatty acids in inflammation.^51^^,^^52^ Circulating monocytes can take up postprandial triglyceride-rich lipoproteins^17^ and increase lipid accumulation independent of lipoprotein lipase.^53^ Future research is needed to identify which lipoprotein subfractions may function as the major driver for monocyte lipid accumulation and phenotypic changes in hypertriglyceridemia and metabolic syndrome.

### Study Limitations

First, we recognize sex differences in ASCVD development. However, we had a relatively small sample size and could not stratify the data according to sex. Second, we examined only a limited number of monocyte markers and did not comprehensively phenotype monocytes in terms of transcriptional reprogramming. Third, our studies included only short-term interventions. The potential effects of long-term dietary interventions on monocytes remain to be determined in future studies. Furthermore, the diets that we used were different not only in saturated fat but also in total fat and cholesterol content. Given that diets rich in saturated fat are usually high in cholesterol in most popular Western diets, it would be difficult to keep cholesterol content low in HSFD. Nonetheless, it will be important in future studies to examine potentially differential effects on monocytes of diets with equal amounts of total fat and cholesterol but different in fat composition, such as HSFD compared with diets high in monounsaturated fat and/or polyunsaturated fat (including n-3 polyunsaturated fatty acids). Despite the increasing popularity of a high-fat diet in metabolic syndrome, our current study showing the adverse effects on monocyte phenotypes with HSFD vs LSFD, along with the increases in LDL-C, in patients with metabolic syndrome and hypertriglyceridemia provides additional evidence for caution regarding the popular meat-based HSFD that is usually also high in cholesterol. In addition, our studies showing no differences in fasting triglyceride levels, but differences in postprandial triglyceride levels, between HSFD and LSFD highlight the relevance of performing postprandial studies when evaluating the potential of triglyceride-modulating interventions.

## Conclusions

The current study shows that in humans with hypertriglyceridemia and metabolic syndrome, monocytes respond differently to a short-term intervention with LSFD or HSFD. After LSFD vs HSFD, monocytes had less lipid accumulation and reduced surface levels of several molecules mediating adhesion, migration, and lipid uptake, with decreases in adhesion to vascular cell adhesion molecule-1 and oxLDL uptake ex vivo. Plasma levels of triglycerides, LDL-TG, RLP-C, apoCIII, and saturated fatty acids, but not total cholesterol, were positively associated with monocyte lipid accumulation and surface levels of CD11c, CCR2, and CD36. These findings highlight the importance of monocyte lipid accumulation and phenotypic changes as a possible link between dietary fat content and composition and the risk for ASCVD in patients with hypertriglyceridemia and metabolic syndrome.

Perspectives **COMPETENCY IN MEDICAL KNOWLEDGE**: Hypertriglyceridemia and metabolic syndrome increase the risk for ASCVD. Consumption of high amounts of saturated fat can increase and reduction of saturated fat consumption can decrease plasma levels of triglycerides and may therefore affect risk for ASCVD. The mechanisms whereby hypertriglyceridemia increases ASCVD risk are not fully understood but may include inflammation and immune cells, including monocytes, which play crucial roles in atherosclerosis. Our current studies showed that in patients with hypertriglyceridemia and metabolic syndrome, short-term LSFD compared with HSFD induced lower plasma levels of postprandial triglycerides and LDL-TG, lower fasting and postprandial total cholesterol and LDL-C, and a lower percentage of saturated fatty acids. Along with these changes, LSFD vs HSFD decreased monocyte intracellular lipid accumulation and reduced monocyte adhesion and oxLDL uptake. These findings highlight the importance of dietary fat content and composition for monocyte phenotypes and possibly ASCVD risk in patients with hypertriglyceridemia and metabolic syndrome.

**TRANSLATIONAL OUTLOOK**: Recently, high-fat, high-protein, but low-carbohydrate, diets have gained popularity in patients with metabolic syndrome, but how diet interventions may affect the risk for ASCVD remains incompletely understood. Our current study showing the adverse effects on monocyte phenotypes with HSFD vs LSFD, along with the increases in LDL-C, in patients with metabolic syndrome and hypertriglyceridemia provides evidence for caution and invites further studies regarding the popular meat-based HSFD that is usually also high in cholesterol.

## Funding Support and Author Disclosures

This work was supported by National Institutes of Health grants R01HL098839, R01DK121348, R01AG065197 (Dr Wu), and R01AI047294 (Dr Simon) and American Heart Association grant 968367 (Dr Wu). Drs Peng, Ni, and Zhang were partially supported by scholarship from the Chinese Scholarship Council. Dr Hoogeveen has received a research grant from Denka Seiken, which provided reagents for small dense LDL-C, RLP-C, and LDL-TG measurements but had no role in the design, analysis, or data interpretation of this study. All other authors have reported that they have no relationships relevant to the contents of this paper to disclose.
