# Supplementary material for: Dietary Effects on Monocyte Phenotypes in Subjects With Hypertriglyceridemia and Metabolic Syndrome
Source: JACC Basic Transl Sci. 2023 Apr 26;8(5):460–75. doi: 10.1016/j.jacbts.2023.02.011 (PMC10264566; doi:10.1016/j.jacbts.2023.02.011)
Supplement: Supplemental Figures 1-4 and Supplemental Tables 1-3 [file mmc1.pdf]

Supplemental Table 1. Antibodies used in the flow cytometry experiments.

Marker	Clone	Company
CCR2	K036C2	BioLegend
CCR5	J418F1	BioLegend
CD11a	m24	BioLegend
CD11c	3.9	BioLegend
CD14	RMO52	Beckman Coulter
CD16	3G8	Beckman Coulter
CD36	FA6.152	Beckman Coulter
CD62L	DREG56	Beckman Coulter
CD81	1D6-CD81	eBioscience
CX3CR1	2A9-1	BioLegend
HLA-DR	Immu-357	Beckman Coulter
LRP1 (CD91)	A2MR-α2	BD Biosciences
SRA	351615	R&D Systems
VLA4	9F10	BioLegend

Supplemental Table 2. Flow cytometric analysis protocols

Protocol	PE	KO	PECy7	V450	PerCPcy5.5	APC	FITC	APC-AF750	APC-Cy7
1	SRA	CD14	CD16		CCR2	CX3CR1	CD36	CD62L	
2	VLA4	CD14	CD16	CD11c		CD81	CD11a	HLA-DR	
3	CD91	CD14	CD16				Nile red		CCR5
4	DiI-oxLDL	CD14	CD16						Viability dye

Supplemental Table 3. Plasma levels of fatty acids and GlycA examined by nuclear magnetic resonance

Fatty acids and GlycA	HSFD				LSFD				P value in 4-day diet phase	P value in postprandial phase
	D1	T0	T4	T6	D1	T0	T4	T6		
Total-FA, mmol/L	13.6±0.67	13.7±0.72	16.3±0.73	15.4±0.65	13.8±0.93	13.5±0.63	16.2±1.02	14.8±0.76	Diet = 0.86<br>Time = 0.77<br>Diet:Time = 0.46	Diet = 0.65<br>Time <0.001<br>Diet:Time = 0.71
Unsaturation, degree	1.34±0.02	1.33±0.02	1.28±0.02	1.3±0.02	1.36±0.02	1.36±0.02	1.33±0.02	1.36±0.02	Diet = 0.019<br>Time = 0.62<br>Diet:Time = 0.81	Diet = 0.008<br>Time <0.001<br>Diet:Time <0.001
Omega-3, mmol/L	0.50±0.04	0.48±0.04	0.57±0.06	0.52±0.05	0.51±0.05	0.53±0.04	0.63±0.05	0.59±0.04	Diet = 0.80<br>Time = 0.37<br>Diet:Time = 0.20	Diet = 0.14<br>Time <0.001<br>Diet:Time = 0.77
Omega-6, mmol/L	4.96±0.13	4.98±0.16	5.44±0.18	5.27±0.18	5.19±0.19	5.04±0.13	5.66±0.24	5.38±0.18	Diet = 0.22<br>Time = 0.88<br>Diet:Time = 0.34	Diet = 0.92<br>Time = 0.001<br>Diet:Time = 0.63
PUFA, mmol/L	5.46±0.15	5.46±0.18	6.01±0.20	5.78±0.20	5.7±0.22	5.57±0.15	6.3±0.27	5.97±0.2	Diet = 0.26<br>Time = 1.00<br>Diet:Time = 0.53	Diet = 0.74<br>Time <0.001<br>Diet:Time = 0.68
MUFA, mmol/L	3.59±0.27	3.58±0.29	4.57±0.25	4.24±0.23	3.58±0.36	3.59±0.25	4.62±0.37	4.06±0.28	Diet = 0.73<br>Time = 0.91<br>Diet:Time = 0.87	Diet = 0.87<br>Time <0.001<br>Diet:Time = 0.50
SFA, mmol/L	4.5±0.29	4.63±0.29	5.75±0.32	5.34±0.27	4.53±0.38	4.34±0.27	5.31±0.42	4.76±0.32	Diet = 0.93<br>Time = 0.39<br>Diet:Time = 0.12	Diet = 0.22<br>Time <0.001<br>Diet:Time = 0.39
LA, mmol/L	3.91±0.12	3.89±0.17	4.33±0.20	4.07±0.19	4.14±0.18	3.95±0.13	4.55±0.25	4.25±0.19	Diet = 0.19<br>Time = 0.89<br>Diet:Time = 0.31	Diet = 0.92<br>Time = 0.002<br>Diet:Time = 0.68
DHA, mmol/L	0.18±0.01	0.18±0.01	0.18±0.02	0.17±0.02	0.19±0.01	0.20±0.01	0.22±0.02	0.20±0.01	Diet = 0.66<br>Time = 0.79	Diet = 0.15<br>Time = 0.14

									Diet:Time = 0.28	Diet:Time = 0.11
Omega-3, %	3.69±0.25	3.54±0.28	3.45±0.28	3.32±0.29	3.69±0.25	3.93±0.21	3.89±0.20	4.0±0.21	Diet = 0.98<br>Time = 0.14<br>Diet:Time = 0.008	Diet = 0.022<br>Time = 0.003<br>Diet:Time <0.001
Omega-6, %	37.5±1.11	37.2±0.96	33.8±0.81	34.6±0.76	38.7±1.15	37.9±0.93	35.6±0.93	36.9±0.85	Diet = 0.041<br>Time = 0.5<br>Diet:Time = 0.46	Diet = 0.14<br>Time <0.001<br>Diet:Time = 0.003
PUFA, %	41.1±1.16	40.7±1.03	37.3±0.81	38±0.77	42.4±1.18	41.9±0.95	39.5±0.90	40.9±0.85	Diet = 0.048<br>Time = 0.41<br>Diet:Time = 0.87	Diet = 0.046<br>Time <0.001<br>Diet:Time <0.001
MUFA, %	26±0.71	25.7±0.71	27.8±0.46	27.4±0.49	25.2±0.72	26.3±0.67	28.1±0.54	27.2±0.52	Diet = 0.023<br>Time = 0.31<br>Diet:Time = 0.002	Diet = 0.15<br>Time <0.001<br>Diet:Time = 0.008
SFA, %	32.8±0.58	33.6±0.46	34.9±0.55	34.6±0.43	32.4±0.60	31.9±0.58	32.4±0.63	31.9±0.55	Diet = 0.38<br>Time = 0.083<br>Diet:Time = 0.005	Diet <0.001<br>Time <0.001<br>Diet:Time = 0.003
LA, %	29.5±0.98	28.8±0.87	26.7±0.8	26.6±0.74	30.8±1.01	29.7±0.85	28.4±0.91	29±0.79	Diet = 0.058<br>Time = 0.27<br>Diet:Time = 0.47	Diet = 0.22<br>Time <0.001<br>Diet:Time = 0.011
DHA, %	1.39±0.12	1.4±0.12	1.13±0.12	1.09±0.11	1.41±0.11	1.52±0.09	1.39±0.08	1.41±0.08	Diet = 0.74<br>Time = 0.79<br>Diet:Time = 0.19	Diet = 0.14<br>Time <0.001<br>Diet:Time <0.001

PUFA/MUFA	1.62±0.08	1.62±0.08	1.35±0.05	1.4±0.05	1.73±0.09	1.62±0.07	1.42±0.05	1.52±0.05	Diet = 0.021<br>Time = 0.98<br>Diet:Time = 0.047	Diet = 0.89<br>Time <0.001<br>Diet:Time <0.001
Omega-6/Omega-3	10.8±0.73	11.3±0.73	10.7±0.71	11.6±0.92	11.3±0.84	10.2±0.67	9.69±0.75	9.78±0.72	Diet = 0.23<br>Time = 0.20<br>Diet:Time = 0.005	Diet = 0.010<br>Time <0.001<br>Diet:Time = 0.007
GlycA, mmol/L	0.91±0.04	0.92±0.04	0.996±0.03	0.96±0.04	0.94±0.05	0.95±0.04	1.03±0.04	1.0±0.05	Diet = 0.34<br>Time = 0.53<br>Diet:Time = 0.85	Diet = 0.56<br>Time = 0.003<br>Diet:Time = 0.75

Data are presented as mean ± SEM. Time points: day 1 fasting (D1); day 5 fasting (T0) and 4 hours (T4) and 6 hours (T6) after test meal.

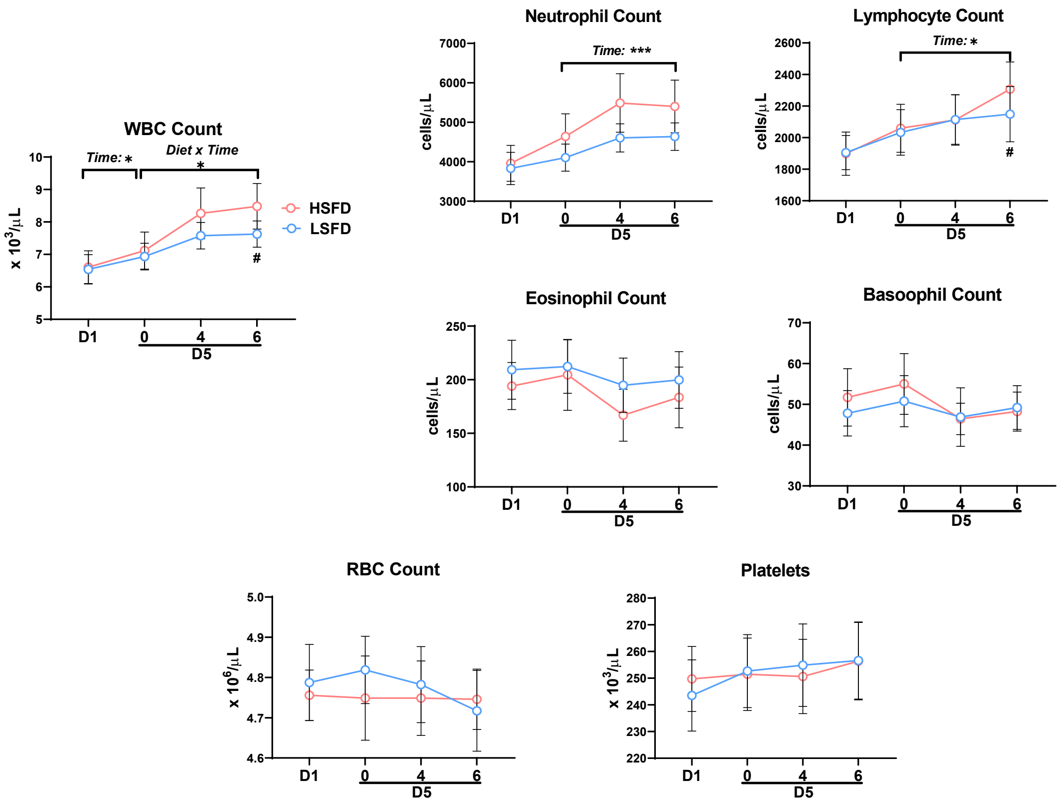

Supplemental Figure 1. Complete blood counts. Counts of total white blood cell (WBC), neutrophils, lymphocytes, eosinophils, basophils, red blood cells (RBC), and platelets. Data are presented as mean ± SEM. *P<0.05, ***P<0.001 for main effects; #P<0.05 post hoc test for pairwise comparisons between diets at indicated time points (day 1 [D1] fasting; day 5 [D5] fasting [0] and 4 hours [4] and 6 hours [6] after a test meal).

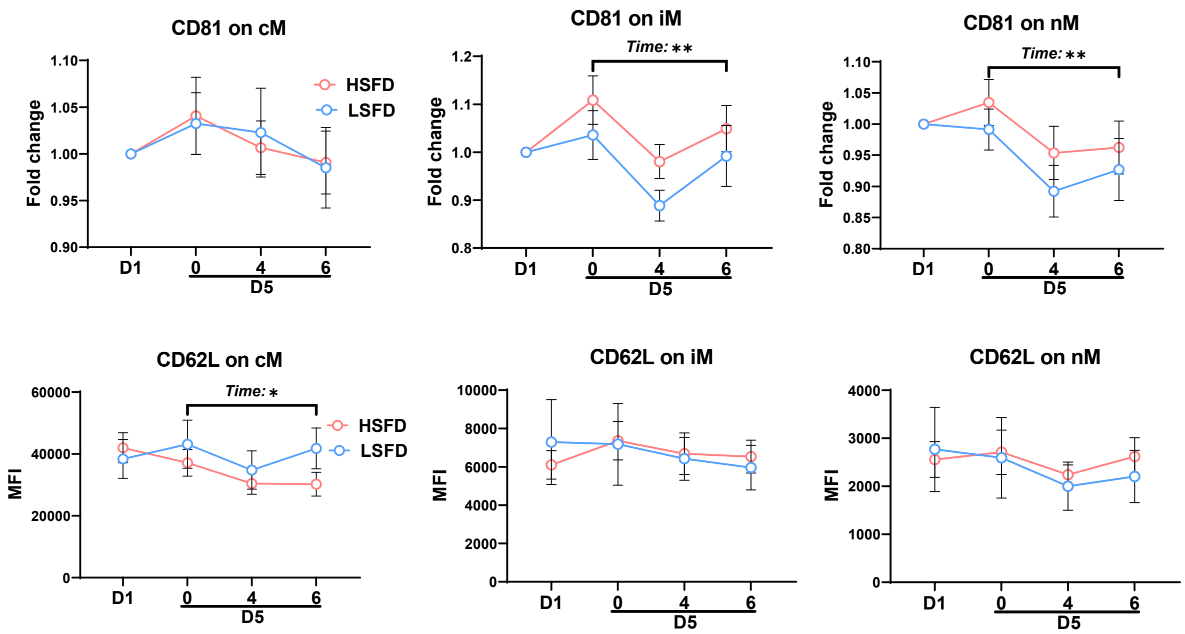

Supplemental Figure 2. Adhesion molecules. Expression levels of adhesion molecules including CD81 and CD62L on monocyte subsets examined by flow cytometry. MFI: mean fluorescence intensity. Data are presented as mean ± SEM. *P<0.05, **P<0.01 for main effects.

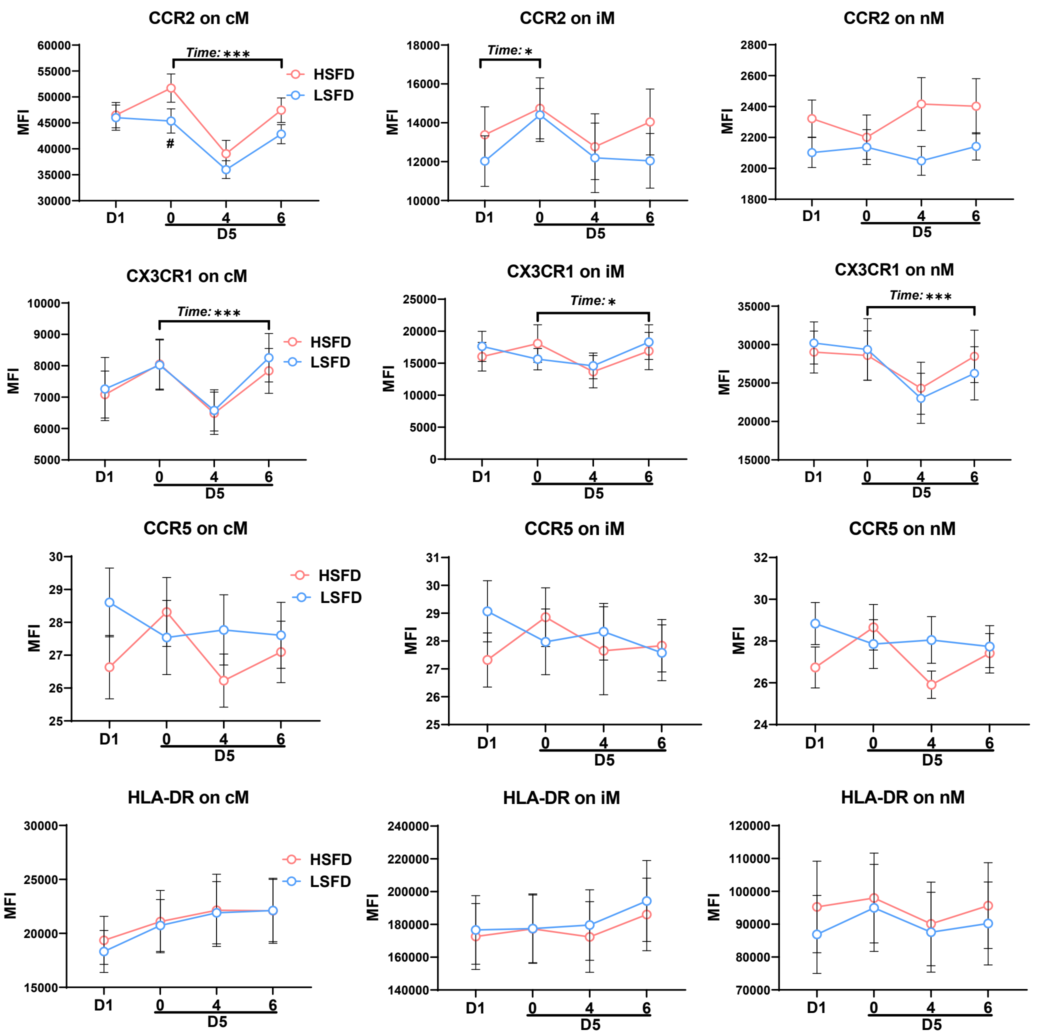

Supplemental Figure 3. Chemokine receptors and HLA-DR. Expression levels, indicated by MFI, of chemokine receptors and HLA-DR on monocyte subsets examined by flow cytometry. Data are presented as mean ± SEM. *P<0.05, ***P<0.001 for main effects; #P<0.05 post hoc test for pairwise comparisons between diets at indicated time points.

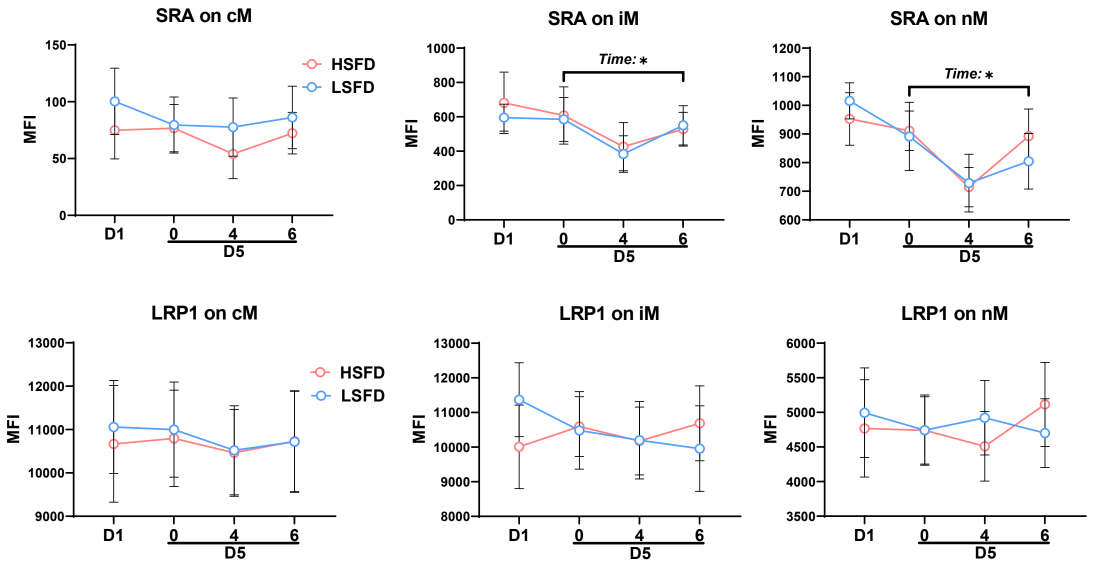

Supplemental Figure 4. Scavenger receptor A (SRA) and LDL receptor–related protein 1 (LRP1). Expression levels, indicated by MFI, of SRA and LRP1 on monocyte subsets examined by flow cytometry. Data are presented as mean ± SEM. *P<0.05 for main effects.
